# Supplementary material for: Comparative interactome analysis of α-arrestin families in human and Drosophila
Source: eLife. 2024 Jan 25;12:RP88328. doi: 10.7554/eLife.88328 (PMC10945707; doi:10.7554/eLife.88328)
Supplement: Supplementary file 12. — The STR analysis report for the HeLa cell line and mycoplasma test results for all cell lines utilized in this study are provided as distinct files. Additionally, the genetic and health monitoring report for the C5BL/6 mouse strain from KOATECH is included. [file elife-88328-supp12.zip › Genetic Monitoring Report (mouse).pdf]

Genetic Monitoring Report

Test Report Date: April 2023

inotiv
analyze. answer. advance.

Location: Koatech	Genetic Monitoring Results			Species: Mouse	
Model	Most Recent Test Date	Recent Results Tested/Passed		Number of SNP Markers Tested	
BALB/cAnNHsd	1-Apr-23	5 / 5		48	
C57BL/6NHsd	1-Apr-23	5 / 5		48	
CBA/JCrHsd	1-Apr-23	5 / 5		48	
DBA/2NHsd	1-Apr-23	5 / 5		48	

Testing Laboratory: TransnetYX Cordova,TN

Inbred strains have been tested using a custom panel of 48 Single Nucleotide Polymorphism (SNP) markers.

Sheri Church

Sheri Church, PhD
Global Geneticist

inotivco.com
8520 Allison Pointe Boulevard, Suite 400, Indianapolis, IN 46250
