# Supplementary material for: Comparative interactome analysis of α-arrestin families in human and Drosophila
Source: eLife. 2024 Jan 25;12:RP88328. doi: 10.7554/eLife.88328 (PMC10945707; doi:10.7554/eLife.88328)
Supplement: Supplementary file 12. — The STR analysis report for the HeLa cell line and mycoplasma test results for all cell lines utilized in this study are provided as distinct files. Additionally, the genetic and health monitoring report for the C5BL/6 mouse strain from KOATECH is included. [file elife-88328-supp12.zip › Health Monitoring Report (mouse).pdf]

Health Monitoring Report

ENVIGO

Facility	Barrier	Species
KOATECH	A103 Hsd:ICR(CD-1®)	Mouse

Viruses	Latest Test Date	Latest Results	Test Method	Historical Results*
Ectromelia virus	18.07.23	0/3	Bead	0/21
Hantavirus	17.01.23	0/3	Bead	0/6
Lactate dehydrogenase elevating virus	17.01.23	0/3	ELISA	0/6
Lymphocytic choriomeningitis virus	18.07.23	0/3	Bead	0/21
Minute virus of mice	18.07.23	0/3	Bead	0/21
Mouse adenovirus type 1 (MAd FL)	18.07.23	0/3	Bead	0/21
Mouse adenovirus type 2 (MAd K87)	18.07.23	0/3	Bead	0/21
Mouse cytomegalovirus	17.01.23	0/3	Bead	0/6
Mouse hepatitis virus	18.07.23	0/3	Bead	0/21
Mouse K-virus	17.01.23	0/3	ELISA	0/6
Mouse Norovirus	18.07.23	0/3	Bead	0/21
Mouse parvovirus	18.07.23	0/3	Bead	0/21
Mouse polyoma virus	17.01.23	0/3	ELISA	0/6
Mouse rotavirus (EDIM)	18.07.23	0/3	Bead	0/21
Mouse thymic virus	17.01.23	0/3	IFA	0/6
Pneumonia virus of mice	18.07.23	0/3	Bead	0/21
Reovirus type 3 (Reo 3)	18.07.23	0/3	Bead	0/21
Sendai Virus	18.07.23	0/3	Bead	0/21
Theiler's murine encephalomyelitis virus	18.07.23	0/3	Bead	0/21
**Bacteria, Mycoplasma and Fungi**				
Bordetella bronchiseptica	17.01.23	0/3	RT-PCR	0/6
CAR Bacillus	17.01.23	0/3	ELISA	0/6
Citrobacter rodentium	18.07.23	0/3	RT-PCR	0/21
Clostridium piliforme	18.07.23	0/3	Bead	0/21
Corynebacterium kutscheri	18.07.23	0/3	RT-PCR	0/21
Dermatophytes	17.01.23	0/3	Culture	0/6
Helicobacter bilis	18.07.23	0/3	RT-PCR	0/21
Helicobacter hepaticus	18.07.23	0/3	RT-PCR	0/21
Klebsiella oxytoca	18.07.23	0/3	RT-PCR	0/21
Klebsiella pneumoniae	18.07.23	0/3	RT-PCR	0/21
Mycoplasma pulmonis	18.07.23	0/3	Bead	0/21
Pasteurella multocida	18.07.23	0/3	RT-PCR	0/21
Pasteurella pneumotropica	18.07.23	0/3	RT-PCR	0/21
Pneumocystis murina	17.01.23	0/3	RT-PCR	0/6
Pseudomonas aeruginosa	18.07.23	0/3	RT-PCR	0/21
Salmonella spp	18.07.23	0/3	RT-PCR	0/21
Streptobacillus moniliformis	18.07.23	0/3	RT-PCR	0/21
Streptococci Beta-haemolytic Group A	18.07.23	0/3	RT-PCR	0/21
Streptococci Beta-haemolytic Group B	18.07.23	0/3	RT-PCR	0/21
Streptococci Beta-haemolytic Group C	18.07.23	0/3	RT-PCR	0/21
Streptococci Beta-haemolytic Group G	18.07.23	0/3	RT-PCR	0/21
Streptococcus pneumoniae	18.07.23	0/3	RT-PCR	0/21
**Parasites**				
Encephalitozoon cuniculi	18.07.23	0/3	ELISA	0/21

Report Reference: 23-7337, Report Printed: July 26, 2023, Barrier Populated: October 26, 2018
Data are expressed as number positive/number tested. *Cumulative data up to 18 months.
All testing is performed by Envigo using standard operating procedures.
Results are controlled through a secure LIMS and released on the approval of the Laboratory Services Manager.

Envigo + Phone:+44 (0)1530 225157 + Fax:+44 (0)1530 222807 + rmstechnicalservices.eu@envigo.com + envigo.com

Health Monitoring Report

Facility	Barrier	Species
KOATECH	A103 Hsd:ICR(CD-1®)	Mouse

Ectoparasites				
Myobia/Radfordia	18.07.23	0/3	RT-PCR	0/21
Myocoptes musculinus	18.07.23	0/3	RT-PCR	0/21

Endoparasites				
Aspiculuris tetraptera	18.07.23	0/3	RT-PCR	0/21
Chilomastix spp	18.07.23	0/3	RT-PCR	0/21
Entamoeba muris	18.07.23	0/3	RT-PCR	0/21
Giardia muris	18.07.23	0/3	RT-PCR	0/21
Spironucleus muris	18.07.23	0/3	RT-PCR	0/21
Syphacia muris	18.07.23	0/3	RT-PCR	0/21
Syphacia obvelata	18.07.23	0/3	RT-PCR	0/21
Tritrichomonas muris	18.07.23	0/3	RT-PCR	0/21

Envigo + Phone:+44 (0)1530 225157 + Fax:+44 (0)1530 222807 + rmstechnicalservices.eu@envigo.com + envigo.com

Health Monitoring Report

ENVIGO

Facility	Barrier	Species
KOATECH	A205 C57BL/6NHsd/BALB/cAnHsd	Mouse

Viruses	Latest Test Date	Latest Results	Test Method	Historical Results*
Ectromelia virus	18.07.23	0/2	Bead	0/14
Hantavirus	17.01.23	0/2	Bead	0/4
Lactate dehydrogenase elevating virus	17.01.23	0/2	ELISA	0/4
Lymphocytic choriomeningitis virus	18.07.23	0/2	Bead	0/14
Minute virus of mice	18.07.23	0/2	Bead	0/14
Mouse adenovirus type 1 (MAd FL)	18.07.23	0/2	Bead	0/14
Mouse adenovirus type 2 (MAd K87)	18.07.23	0/2	Bead	0/14
Mouse cytomegalovirus	17.01.23	0/2	Bead	0/4
Mouse hepatitis virus	18.07.23	0/2	Bead	0/14
Mouse K-virus	17.01.23	0/2	ELISA	0/4
Mouse Norovirus	18.07.23	0/2	Bead	0/14
Mouse parvovirus	18.07.23	0/2	Bead	0/14
Mouse polyoma virus	17.01.23	0/2	ELISA	0/4
Mouse rotavirus (EDIM)	18.07.23	0/2	Bead	0/14
Mouse thymic virus	17.01.23	0/2	IFA	0/4
Pneumonia virus of mice	18.07.23	0/2	Bead	0/14
Reovirus type 3 (Reo 3)	18.07.23	0/2	Bead	0/14
Sendai Virus	18.07.23	0/2	Bead	0/14
Theiler's murine encephalomyelitis virus	18.07.23	0/2	Bead	0/14
**Bacteria, Mycoplasma and Fungi**				
Bordetella bronchiseptica	17.01.23	0/2	RT-PCR	0/4
CAR Bacillus	17.01.23	0/2	ELISA	0/4
Citrobacter rodentium	18.07.23	0/2	RT-PCR	0/14
Clostridium piliforme	18.07.23	0/2	Bead	0/14
Corynebacterium kutscheri	18.07.23	0/2	RT-PCR	0/14
Dermatophytes	17.01.23	0/2	Culture	0/4
Helicobacter bilis	18.07.23	0/2	RT-PCR	0/14
Helicobacter hepaticus	18.07.23	0/2	RT-PCR	0/14
Klebsiella oxytoca	18.07.23	0/2	RT-PCR	0/14
Klebsiella pneumoniae	18.07.23	0/2	RT-PCR	0/14
Mycoplasma pulmonis	18.07.23	0/2	Bead	0/14
Pasteurella multocida	18.07.23	0/2	RT-PCR	0/14
Pasteurella pneumotropica	18.07.23	0/2	RT-PCR	0/14
Pneumocystis murina	17.01.23	0/2	RT-PCR	0/4
Pseudomonas aeruginosa	18.07.23	0/2	RT-PCR	0/14
Salmonella spp	18.07.23	0/2	RT-PCR	0/14
Streptobacillus moniliformis	18.07.23	0/2	RT-PCR	0/14
Streptococci Beta-haemolytic Group A	18.07.23	0/2	RT-PCR	0/14
Streptococci Beta-haemolytic Group B	18.07.23	0/2	RT-PCR	0/14
Streptococci Beta-haemolytic Group C	18.07.23	0/2	RT-PCR	0/14
Streptococci Beta-haemolytic Group G	18.07.23	0/2	RT-PCR	0/14
Streptococcus pneumoniae	18.07.23	0/2	RT-PCR	0/14
**Parasites**				
Encephalitozoon cuniculi	18.07.23	0/2	ELISA	0/14

Report Reference: 23-7341, Report Printed: July 26, 2023, Barrier Populated: October 26, 2018
Data are expressed as number positive/number tested. *Cumulative data up to 18 months.
All testing is performed by Envigo using standard operating procedures.
Results are controlled through a secure LIMS and released on the approval of the Laboratory Services Manager.

Health Monitoring Report

Facility	Barrier	Species
KOATECH	A205 C57BL/6NHsd/BALB/cAnHsd	Mouse

**Ectoparasites**				
Myobia/Radfordia	18.07.23	0/2	RT-PCR	0/14
Myocoptes musculinus	18.07.23	0/2	RT-PCR	0/14
**Endoparasites**				
Aspiculuris tetraptera	18.07.23	0/2	RT-PCR	0/14
Chilomastix spp	18.07.23	0/2	RT-PCR	0/14
Entamoeba muris	18.07.23	0/2	RT-PCR	0/14
Giardia muris	18.07.23	0/2	RT-PCR	0/14
Spironucleus muris	18.07.23	0/2	RT-PCR	0/14
Syphacia muris	18.07.23	0/2	RT-PCR	0/14
Syphacia obvelata	18.07.23	0/2	RT-PCR	0/14
Tritrichomonas muris	18.07.23	0/2	RT-PCR	0/14

Report Reference: 23-7341, Report Printed: July 26, 2023, Barrier Populated: October 26, 2018
Data are expressed as number positive/number tested. *Cumulative data up to 18 months.
All testing is performed by Envigo using standard operating procedures.
Results are controlled through a secure LIMS and released on the approval of the Laboratory Services Manager.
