# Supplementary material for: Comparative interactome analysis of α-arrestin families in human and Drosophila
Source: eLife. 2024 Jan 25;12:RP88328. doi: 10.7554/eLife.88328 (PMC10945707; doi:10.7554/eLife.88328)
Supplement: Supplementary file 12. — The STR analysis report for the HeLa cell line and mycoplasma test results for all cell lines utilized in this study are provided as distinct files. Additionally, the genetic and health monitoring report for the C5BL/6 mouse strain from KOATECH is included. [file elife-88328-supp12.zip › HeLa STR anaylsis report (ATCC#CCL-2).pdf]

Cell line STR

Analysis Result Report

INFORMATION

Order No.	HC00683860
Date	2023. 04. 14
Sample Type	Cell line

METHOD

- The AmpFlSTR® Identifiler® PCR Amplification Kit is a short tandem repeat (STR) multiplex assay that amplifies 15 tetranucleotide repeat loci and the Amelogenin gender-determining marker in a single PCR amplification:

  - All thirteen of the required loci for the Combined DNA Index System (CODIS) (Budowle et al., 1998).
  - Two additional loci, D2S1338 and D19S433.

- We use Applied Biosystems® 3730/3730xl DNA Analyzer and analyze data using GeneMapper ID v3.2. For reliability, Positive (DNA9947A) and Negative are used together during the test.

- We do experiment based on the Genotype Identification Kit of ABI which is international standard method, and we also obtained a method using PowerPlex21 of Promega to secure diversity.

REFERENCE

- Budowle, B. et al. 1998. CODIS and PCR Based Short Tandem Repeat Loci: Law Enforcement Tools. Second European Symposium on Human Identification. 73–88.
- Holt, C., Stauffer, C., Wallin, J., Lazaruk, L., Nguyen, T., Budowle, B., and Walsh, P. 2000. Practical applications of genotypic Surveys for forensic STR testing. Forensic Sci.
- Butler, J.M. 2005. Forensic DNA Typing. Burlington, MA:Elsevier Academic Press.

PROCESS

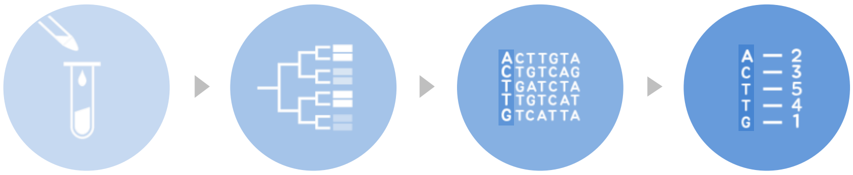

DNA extraction → PCR amplification → Sequencing analysis → Alleles Profile (STR analysis)

NOTE

- **Cell line authentication?**

  Cell line authentication Service is genetic analyzing service using Multiplex-PCR Kit which is comprised of STR(Short Tandem Repeat) loci.

- **STR Marker Information (AmpFISTR®Identifiler® PCR Amplification KIT)**

STR Locus	Label	Size Range	Chromosomal Location	Repeat Sequence 5'→3'
D8S1179	6-FAM	123-170	8q24.13	(TCTA)(TCTG)
D21S11		185-239	21q21.1	(TCTA)(TCTG)
D7S820		255-291	7q21.11	GATA
CSF1PO		305-342	5q33.1	TAGA
D3S1358	VIC	112-140	3p21.31	(TCTA)(TCTA)
TH01		163-202	11p15.5	TCAT
D13S317		217-245	13q31.1	TATC
D16S539		252-292	16q24.1	GATC
D2S1338		307-359	2q35	(TGCC)(TTCC)
Amelogenin	PET	107,113	XP22.22/YP11.2	NA
D5S818		134-172	5q23.2	AGAT
FGA		215-355	4q31.3	CTTT
D19S433	NED	102-135	19q12	AAGG
vWA		155-207	12q13.31	(TCTG)(TCTA)
TPOX		222-250	2p25.3	GAAT
D18S51		232-345	18q21.33	AGAA

Humanizing Genomics macrogen | CES CAPILLARY ELECTROPHORESIS SEQUENCING

Cell line authentication

Result Report

Do date.	2023. 04. 14	Method	A-STR Genotyping analysis, 3730XL DNA analyzer
Reagent	AmpFISTR®Identifiler® KIT	Percent Match	100 %

Cell name	HeLa(Reference)		3	
Amelogenin	X	X	X	X
STR Locus	Allele1	Allele2	Allele1	Allele2
D8S1179			12	13
D21S11			27	28
D7S820	8	12	8	12
CSF1PO	9	10	9	10
D3S1358			15	18
TH01	7	7	7	7
D13S317	12	13.3	12	13.3
D16S539	9	10	9	10
D2S1338			17	171
D5S818	11	12	11	12
FGA			18	21
D19S433			13	14
vWA	16	18	16	18
TPOX	8	12	8	12
D18S51			16	16

Interpretation of Results

** Reference : ICLAC - Match Criteria for Human Cell Line Authentication*

1 Is the percent match result in the range 0 - 55% ?

- This result is consistent with the two samples being unrelated (different donors)

2 Is the percent match result in the range 56 - 79% ?

- This result is indeterminant and may need further testing

3 Is the percent match result in the range 80 - 100% ?

- This result is consistent with the two samples being related (same donor)

Yang Y.J.

2023.04.14

Laboratory Director YongJin Yang

*This report is for research purposes only

Raw Data Guide

① Sample name
② STR amplification kit
③ STR marker
④ X axis: Fragment size
⑤ Y axis: Relative fluorescence units (RFU)
⑥ STR repeat number (13 = 13 repeats)
⑦ Decimal point (9.3 = ATGC ATGC …. ATGC ATG)
⑧ sex determining marker (Female: XX / Male: XY)

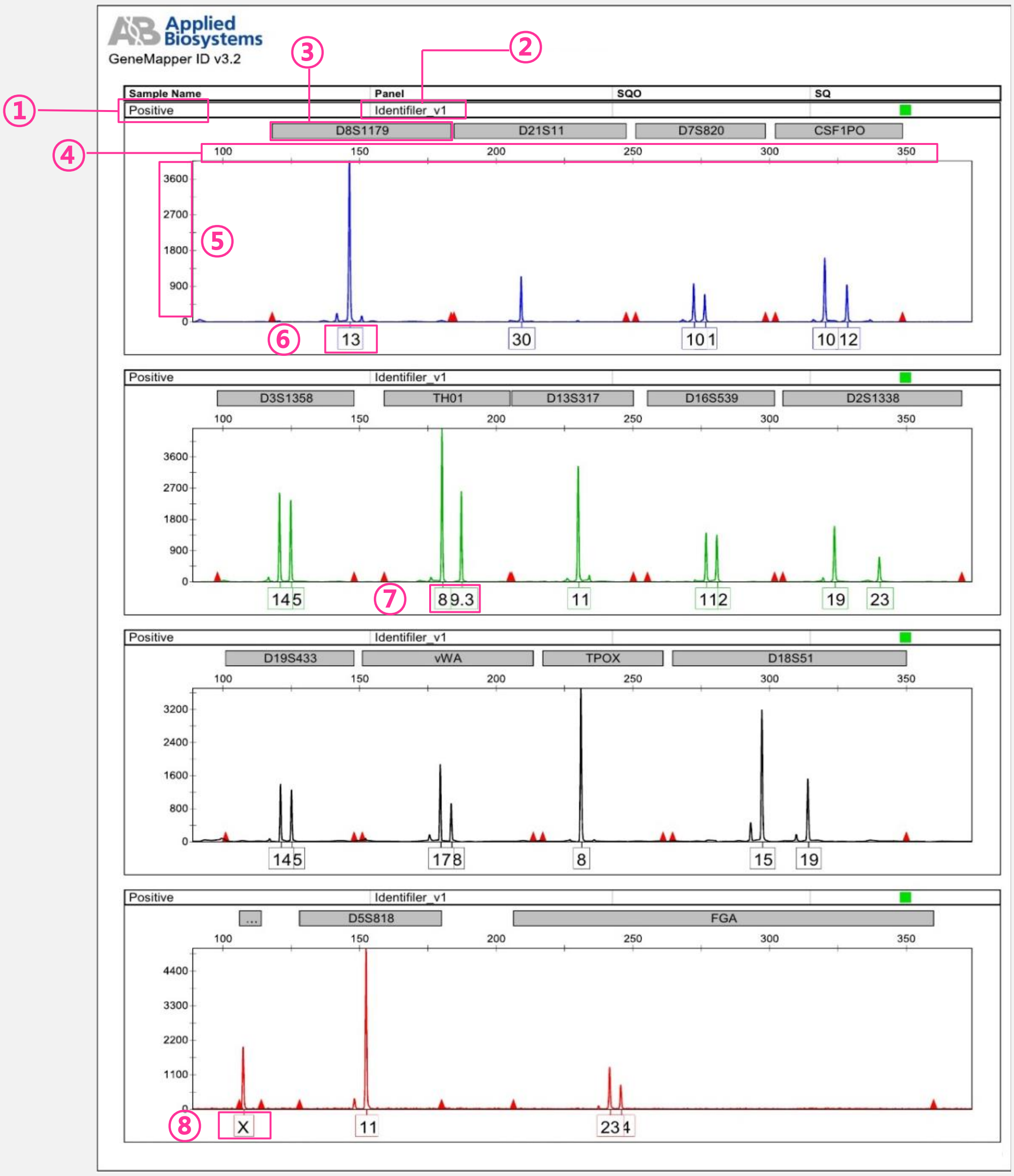

10F, 254, Beotkkot-ro Geumcheon-gu, Seoul, Republic of korea (Gasna-dong, World Meridian I)
TEL : +82-2-2180-7134 / E.mail : hg1427@macrogen.com
