# Supplementary figures and images for: Comparative interactome analysis of α-arrestin families in human and Drosophila
Source: eLife. 2024 Jan 25;12:RP88328. doi: 10.7554/eLife.88328 (PMC10945707; doi:10.7554/eLife.88328)

## Mycoplasma testing

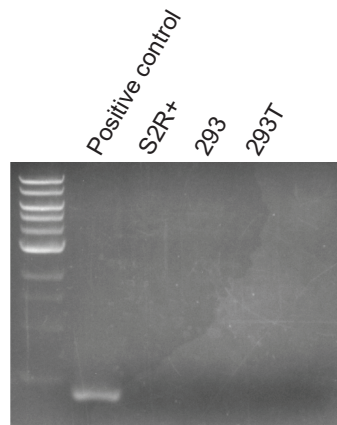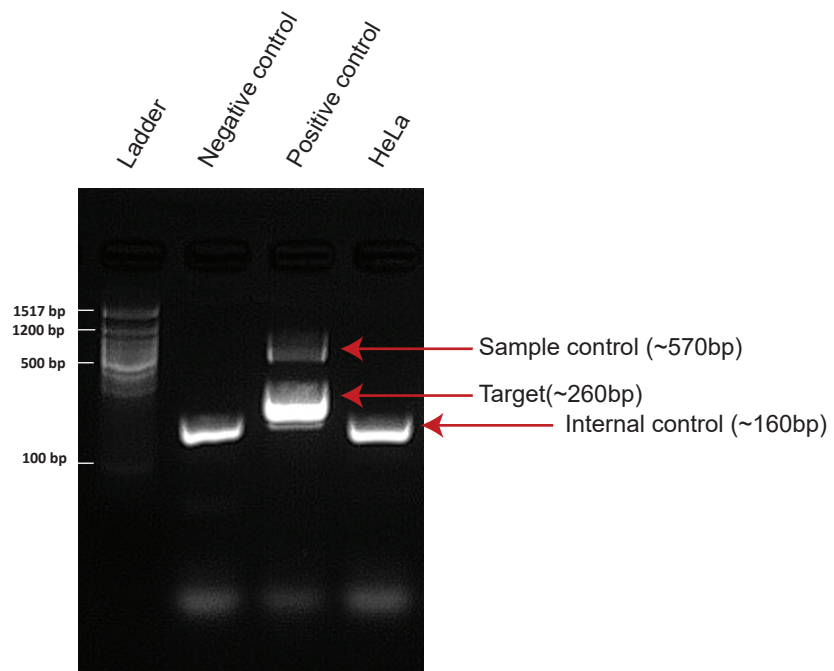

Supplement: Supplementary file 12. — The STR analysis report for the HeLa cell line and mycoplasma test results for all cell lines utilized in this study are provided as distinct files. Additionally, the genetic and health monitoring report for the C5BL/6 mouse strain from KOATECH is included. [file elife-88328-supp12.zip › Mycoplasma test results.pdf]
